# Supplementary material for: Functional phenotyping of genomic variants using joint multiomic single-cell DNA–RNA sequencing
Source: Nat Methods. 2025 Sep 1;22(10):2032–41. doi: 10.1038/s41592-025-02805-0 (PMC12510883; doi:10.1038/s41592-025-02805-0)
Supplement: Supplementary file 1 — Reporting Summary [file 41592_2025_2805_MOESM1_ESM.pdf]

nature portfolio

Corresponding author(s): Lars Steinmetz

Last updated by author(s): Jun 18, 2025

Reporting Summary

Nature Portfolio wishes to improve the reproducibility of the work that we publish. This form provides structure for consistency and transparency in reporting. For further information on Nature Portfolio policies, see our Editorial Policies and the Editorial Policy Checklist.

Statistics

For all statistical analyses, confirm that the following items are present in the figure legend, table legend, main text, or Methods section.

n/a	Confirmed	
☐	☒	The exact sample size (*n*) for each experimental group/condition, given as a discrete number and unit of measurement
☒	☐	A statement on whether measurements were taken from distinct samples or whether the same sample was measured repeatedly
☐	☒	The statistical test(s) used AND whether they are one- or two-sided<br>*Only common tests should be described solely by name; describe more complex techniques in the Methods section.*
☒	☐	A description of all covariates tested
☐	☒	A description of any assumptions or corrections, such as tests of normality and adjustment for multiple comparisons
☐	☒	A full description of the statistical parameters including central tendency (e.g. means) or other basic estimates (e.g. regression coefficient) AND variation (e.g. standard deviation) or associated estimates of uncertainty (e.g. confidence intervals)
☐	☒	For null hypothesis testing, the test statistic (e.g. *F*, *t*, *r*) with confidence intervals, effect sizes, degrees of freedom and *P* value noted<br>*Give P values as exact values whenever suitable.*
☒	☐	For Bayesian analysis, information on the choice of priors and Markov chain Monte Carlo settings
☒	☐	For hierarchical and complex designs, identification of the appropriate level for tests and full reporting of outcomes
☐	☒	Estimates of effect sizes (e.g. Cohen's *d*, Pearson's *r*), indicating how they were calculated

Our web collection on statistics for biologists contains articles on many of the points above.

Software and code

Policy information about availability of computer code

Data collection	SDR-seq was performed using the Tapestri microfluidic device from Mission Bio. Flow cytometry was performed using BD Fortessa instruments running Diva (V9.0.1) software. Illumina sequencers (HiSeq and NextSeq) were used for NGS experiments.
Data analysis	Data analysis was performed using SDRranger (v1.0) to generate count/read matrices from RNA or gDNA NGS data (https://github.com/hawkjo/SDRranger). Code for TAP-seq prediction, generation of custom STAR references and processing of the data is available under https://github.com/DLindenhofer/SDR-seq. Packages used were AnnotationDbi (1.64.1), BiocManager (1.30.25), BiocParallel (1.36.0), biomaRt (2.58.2), Biostrings (2.70.3), BSgenome (1.70.2), BSgenome.Mmusculus.UCSC.mm10 (1.4.3), cardelino (1.4.0), circlize (0.4.16), data.table (1.17.0), dplyr (1.1.4), future.apply (1.11.3), GenomicRanges (1.54.1), ggpattern (1.1.4), ggplot2 (3.5.2), here (1.0.1), knitr (1.50), Matrix (1.6.5), org.Hs.eg.db (3.18.0), patchwork (1.3.0), pcaMethods (1.94.0), pheatmap (1.0.12), purrr (1.0.4), RColorBrewer (1.1.3), readr (2.1.5), readxl (1.4.5), reshape2 (1.4.4), Rmisc (1.5.1), rtracklayer (1.62.0), scales (1.4.0), Seurat (5.3.0), stringr (1.5.1), TAPseq (1.14.1), tibble (3.2.1), tidyr (1.3.1), tidyverse (2.0.0), topGO (2.54.0), STAR (2.7.11a), Python (3.8.0), GATK HaplotypeCaller (4.2.3.0), sinto (0.10.0) and samtools (1.17).

For manuscripts utilizing custom algorithms or software that are central to the research but not yet described in published literature, software must be made available to editors and reviewers. We strongly encourage code deposition in a community repository (e.g. GitHub). See the Nature Portfolio guidelines for submitting code & software for further information.

Data

Policy information about availability of data

All manuscripts must include a data availability statement. This statement should provide the following information, where applicable:

- Accession codes, unique identifiers, or web links for publicly available datasets
- A description of any restrictions on data availability
- For clinical datasets or third party data, please ensure that the statement adheres to our policy

Sequencing data and processed data for non-primary human data is available on GEO under accession number GSE268646. Sequencing data and processed data for primary human data is available on EGA under study number EGAS50000000374 and dataset ID EGAD50000000551. The dataset on EGA is read-only under ega-archive.org/datasets/EGAD50000000551. Access to the data will be granted for appropriate use in research and will be governed by the provisions laid out in the terms contained in the Data Access Agreement. Variant information for WTC-11 human iPSCs was downloaded from UCSC (https://s3-us-west-2.amazonaws.com/downloads.allencell.org/genome-sequence/AH77TTBBXX_DS-229105_GCCAAT_recalibrated.vcf.gz). Candidate cis-regulatory elements (cCRE) for five human iPSC lines (H1, H7, H9, iPS DF 6.9, iPS DF 19.11) were obtained from SCREEN (https://screen.encodeproject.org). iPSCs data for ParseBio data was obtained from https://www.parsebiosciences.com/customer-datasets/multi-omics-approach-for-near-full-length-human-ipsc-transcriptomes-in-cardiomyocyte-models/#download. NIH-3T3 data for TAP-seq primer prediction was obtained from https://www.10xgenomics.com/datasets/500-1-1-mixture-of-human-hek-293-t-and-mouse-nih-3-t-3-cells-3-lt-v-3-1-chromium-x-3-1-low-6-1-0. WTC-11 data for TAP-seq primer prediction and comparison of gene expression variance was obtained from https://www.ebi.ac.uk/biostudies/arrayexpress using accession number E-MTAB-6687. DNA sequences for custom gDNA and RNA references used for alignment were obtained in R using the BSgenome (1.70.2) package utilizing the "BSgenome.Hsapiens.UCSC.hg38" genome" (genome hg38, based on assembly GRCh38.p14 since 2023/01/31).

Research involving human participants, their data, or biological material

Policy information about studies with human participants or human data. See also policy information about sex, gender (identity/presentation), and sexual orientation and race, ethnicity and racism.

Reporting on sex and gender	No sex or gender-based analysis were performed as they were not relevant in this study. Informed consent from every patient was gathered beforehand to collect gender data and was determined based on self-reporting. Gender is reported in the EGA repository in the metadata.
Reporting on race, ethnicity, or other socially relevant groupings	This information was not collected. Therefore not applicable.
Population characteristics	Informed consent from every patient was gathered beforehand regarding age, diagnosis and treatment. None of this metadata is taken in consideration in the data analysis in this manuscript. Diagnosis was either follicular lymphoma (FL1 and FL2) or germinal center subtype diffuse large B-cell lymphoma (GCB1). Age at time of sampling was 59 (Fl1), 74 (Fl2) and 45 (GCB1). Sex was female for FL1 and male for FL2 and GCB1. Ann-Arbor clinical stage at time of sampling was IIIA (Fl1), IVA (Fl2) and IVB (GCB1). Relapse status at time of sampling was diagnosis (FL1 and GCB1) and relapse (Fl2).
Recruitment	Recruitment for this retrospective study was done from suitable biobanked material at University Hospital Heidelberg.
Ethics oversight	The study (S-254/2016) was approved by University of Heidelberg's Ethics Committee. We obtained informed consent from every patient beforehand.

Note that full information on the approval of the study protocol must also be provided in the manuscript.

Field-specific reporting

Please select the one below that is the best fit for your research. If you are not sure, read the appropriate sections before making your selection.

☒ Life sciences ☐ Behavioural & social sciences ☐ Ecological, evolutionary & environmental sciences

For a reference copy of the document with all sections, see nature.com/documents/nr-reporting-summary-flat.pdf

Life sciences study design

All studies must disclose on these points even when the disclosure is negative.

Sample size	No statistical methods were used to predetermine sample sizes. Instead, sample sizes were guided by the technical limitations of the Tapestri microfluidic device, which yields approximately 9,000 cells per run. This throughput is consistent with other widely used single-cell microfluidic platforms, such as 10x Genomics. For each experiment, the number of cells analyzed was chosen to be sufficient for qualitative and comparative assessment of assay performance and biological signal detection in the context o method development.<br><br>The manuscript primarily describes SDR-seq, and the experimental design focuses on demonstrating technical feasibility, robustness, and versatility. Robustness was shown by reproducing the assay across multiple independent runs, yielding reproducible combined single-cell readouts of gDNA and RNA in the same cell. Fixation condition effects were assessed in a multiplexed design within a single run, allowing direct cell-to-cell comparison under identical processing conditions. Primer panel size effects were evaluated in separate SDR-seq runs due to

the need for distinct PCR panels, and sufficient cells were obtained in each run to assess performance and coverage.

In perturbation experiments, the observed low editing efficiency limited the ability to interpret a large number of eQTLs, but the data generated were sufficient to highlight this challenge and inform future optimization. Finally, samples from three B-cell lymphoma patients were processed across two independent SDR-seq runs. The sample size was adequate to demonstrate the applicability of the method , performing differential abundance testing of variants and differential gene expression analysis comparing distinct subclasses of cells within each patient.

Overall, sample sizes were selected to balance the throughput limits of the technology with the goal of establishing method feasibility, reproducibility, and practical use cases. The number of cells and samples per experiment was sufficient to achieve these aims.

Data exclusions	Low quality cells were removed for downstream processing. Detailed thresholds set for each experiment can be found in https://github.com/DLindenhofer/SDR-seq. A second GCB sample was intended to be analyzed in this study. Low viability after thawing and dead cell removal prohibited the inclusion of this sample.
Replication	SDR-seq was shown to work in two different fixation conditions and across different panel sizes, perturbation assays and sample types. All attempts of performing SDR-seq as described in the manuscript were successful. Overall these were 10 independent SDR-seq runs.
Randomization	Not applicable in this study as experiments doing comparative analysis were assayed in a pooled setting.
Blinding	The experimenters were not blinded. Experimental procedures were automated and standardized, and data analyses were carried out predominantly using computational pipelines without manual intervention. The reported results are primarily descriptive and based on objective readouts such as sequencing metrics, read counts, and computationally derived molecular profiles. As such, the experimenter had no opportunity to influence the outcomes.

Reporting for specific materials, systems and methods

We require information from authors about some types of materials, experimental systems and methods used in many studies. Here, indicate whether each material, system or method listed is relevant to your study. If you are not sure if a list item applies to your research, read the appropriate section before selecting a response.

Materials & experimental systems

n/a	Involved in the study
☒	☐ Antibodies
☐	☒ Eukaryotic cell lines
☒	☐ Palaeontology and archaeology
☒	☐ Animals and other organisms
☒	☐ Clinical data
☒	☐ Dual use research of concern
☒	☐ Plants

Methods

n/a	Involved in the study
☒	☐ ChIP-seq
☐	☒ Flow cytometry
☒	☐ MRI-based neuroimaging

Eukaryotic cell lines

Policy information about cell lines and Sex and Gender in Research

Cell line source(s)	The HEK293 line was purchased from ATCC (CRL-3216). The WTC-11 iPSCs (GM25256)were purchased from the Coriell Institute for Medical Research. The NIH-3T3 cell line was purchased from DSMZ (ACC 59)
Authentication	None of the cell lines were independently authenticated.
Mycoplasma contamination	Cell cultures were routinely (every three months) tested and confirmed negative for mycoplasma.
Commonly misidentified lines (See ICLAC register)	No commonly misidentified lines have been used in this study.

Plants

Seed stocks

NA

Novel plant genotypes

NA

Authentication

NA

Flow Cytometry

Plots

Confirm that:

☒ The axis labels state the marker and fluorochrome used (e.g. CD4-FITC).

☒ The axis scales are clearly visible. Include numbers along axes only for bottom left plot of group (a 'group' is an analysis of identical markers).

☒ All plots are contour plots with outliers or pseudocolor plots.

☒ A numerical value for number of cells or percentage (with statistics) is provided.

Methodology

Sample preparation	For flow cytometry analysis iPSCs were prepared in a single cell suspension using Accutase (StemCell Technologies - #07922). This was followed by filtering through a 35 μm cell strainer.
Instrument	BD Fortessa
Software	FACS Diva
Cell population abundance	A minimum of 20000 single cells was analyzed for each condition at each timepoint.
Gating strategy	Single cells were gated using forward and side scatters. Amplifier settiings were chosen to clearly display negative and positive populations. Gating strategies are provided in the Extended Data Fig. 6e directly next to each flow cytometry plot.

☒ Tick this box to confirm that a figure exemplifying the gating strategy is provided in the Supplementary Information.
